# A Machine Learning Model Based on Multi-Phase Contrast-enhanced CT for the Preoperative Prediction of the Muscle-Invasive Status of Bladder Cancer

**DOI:** 10.2174/0115734056377754250304040058

**Published:** 2025-03-17

**Authors:** Xucheng He, Yuqing Chen, Shanshan Zhou, Guisheng Wang, Rongrong Hua, Caihong Li, Yang Wang, Xiaoxia Chen, Ju Ye

**Affiliations:** 1 Department of Radiology, the Third Medical Center, Chinese PLA General Hospital, Beijing100039, China; 2 Department of Radiology, Dongfang Hospital, Beijing University of Chinese Medicine, Beijing100039, China

**Keywords:** Machine learning (ML), Bladder cancer, Contrast-enhanced computer tomography (CECT), Muscle-invasive, Receiver operating characteristic (ROC), Least absolute shrinkage selection operator (LASSO), Uroepithelial carcinoma

## Abstract

**Background:**

Muscle infiltration of bladder cancer has become the most important index to evaluate its prognosis. Machine learning is expected to accurately identify its muscle infiltration status on images.

**Objective:**

This study aimed to establish and validate a machine learning prediction model based on multi-phase contrast-enhanced CT (MCECT) for preoperatively evaluating the muscle-invasive status of bladder cancer.

**Methods:**

A retrospective study was conducted on bladder cancer patients who underwent surgical resection and pathological confirmation by MCECT scans. They were randomly divided into training and testing groups at a ratio of 8:2; we used an independent external testing set for verification. The radiomics features of lesions were extracted from MCECT images and radiomics signatures were established by dual sample T-test and least absolute shrinkage selection operator (LASSO) regression. Afterward, four machine learning classifier models were established. The receiver operating characteristic (ROC) curve, calibration, and decision curve analysis were employed to evaluate the efficiency of the model and analyze diagnostic performance using accuracy, precision, sensitivity, specificity, and F1-score.

**Results:**

The best predictive model was found to have logic regression as the classifier. The AUC value was 0.89 (5-fold cross-validation range 0.83-0.96) in the training group, 0.80 in the test group, and 0.87 in the external testing group. In the testing and external testing group, the diagnostic accuracy, precision, sensitivity, specificity, and F1-score were 0.759, 0.826, 0.863, 0.729, 0.785, and 0.794, 0.755, 0.953, 0.720, and 0.809, respectively.

**Conclusion:**

The machine learning model showed good accuracy in predicting the muscle infiltration status of bladder cancer before surgery.

## 1 INTRODUCTION

Bladder cancer is the second most common urological malignancy worldwide, whose incidence also ranks ninth among all malignancies globally [1]. Uroepithelial carcinoma accounts for approximately 90% of bladder cancer cases and typically presents as multifocal and recurrent; other subtypes are squamous cell carcinoma and adenocarcinoma [2]. The latest research shows that it is more important to judge whether bladder cancer muscle is infiltrated before surgery than pathological grading. According to whether muscle infiltrates bladder cancer, it can be classified as either muscle-invasive bladder cancer (MIBC) or non-muscle-invasive bladder cancer (NMIBC). About 75% of newly diagnosed bladder cancer patients have NMIBC while the remaining patients have MIBC [3, 4]. However, the presence or absence of muscle invasion has a significant impact on the five-year survival rate of patients [5, 6].

Magnetic resonance imaging (MRI) is an important method for locoregional staging of bladder cancer and has proven to be more powerful in differentiating between MIBC and NMIBC, specifically in differentiating T1 from T2 disease, and it may lead to a change in treatment approach in patients at high risk of an invasive tumor [7]. However, MCECT has the advantages of short imaging time, thin scanning layer, wide scanning range, full urography, and equal voxel multi-planar reconstruction and three-dimensional reconstruction; it plays an irreplaceable role in the diagnosis and preoperative evaluation of bladder cancer. Although MRI and multi-sequence CT play an important role in the diagnosis and staging of bladder cancer, it is difficult to diagnose the muscle infiltration of bladder cancer on medical images [8].

Our eyes can recognize little features in an image, while radiomics can extract more features within the image that the naked eye cannot recognize. With the diagnostic and predictive potential, radiomics can also assist doctors in formulating clinical and treatment strategies for patients [9]. Furthermore, machine learning algorithms have been widely used in clinical research. Based on the advantages of machine learning in medicine, more and more studies have innovatively combined machine learning and radiomics in research. There are many algorithms for classification, and the most significant are logic regression and support vector classification (SVC). In previous studies, MRI VI-RADS score has been used as the main method to evaluate muscle invasion of bladder cancer [10]. Currently, there are several methods to predict the pathological grading of bladder cancer based on MCECT and MRI radiomics [11, 12], predict muscle invasion of cancer based on MRI and CT radiomics [13, 14], and predict the depth of muscle invasion of bladder cancer using machine learning for MRI images [15], which have achieved satisfactory results. However, there are few studies on the prediction of muscle invasion by using MCECT to establish multiple machine learning models.

In this study, 4 radiomics models based on machine learning were established for the prediction of bladder cancer’s muscle-invasive status. By evaluating the performance, the best machine learning model was selected to improve the differentiation power. Fig. (**1**) describes the workflow of this study.

## 2 MATERIALS AND METHODS

### 2.1 Patients and CT Data Acquisition

The retrospective data of bladder cancer patients who were operated at our hospital from October 2020 to September 2023 and confirmed by pathology were collected. The inclusion criteria were as follows: a. pathological data confirming the presence of muscle infiltration; b. MCECT images being complete; c. no visible infiltration of connective tissue or lymph node metastasis around the bladder on the image; d. MCECT performed before surgery. The exclusion criteria were as follows: a. CT quality being unsatisfactory, mainly manifested in incomplete sequences and various artifacts in the images; b. missing clinical pathological data; c. connective tissue infiltration or lymph node metastasis around the bladder being visible from the image; d. having undergone cystectomy before the CT scan; e. CT images with lesions smaller than 0.5cm. 148 patients were randomly assigned to the training group (n=118) and the testing group (n=30), with a ratio of 8:2, and the proportion of tumors in the training and experimental groups was kept as equal as possible. At the same time, we introduced an independent external testing set (63 cases) to verify the performance of the model.

Images were scanned using two CT equipment: GE Discovery CT750 HD and Siemens Somatom Definition Flash. All examinations used the CECT protocol. The three phases (plain phase, arterial phase, and venous phase) were selected for radiomics analysis. The detailed acquisition parameters of the image are shown in Table **1**.

### 2.2 Regions of Interest Segmentation and Radiomics Features Extraction

The non-enhanced, arterial, and venous phases’ CT images of all patients were uploaded into the open-source 3D slicer (v.5.0.3). Region of interest (ROI) segmentation was performed by an experienced radiologist blinded to all clinical, pathologic, and follow-up information. ROI segmentation based on MCECT images was independently and manually performed slice by slice throughout the whole tumor.

All images were resampled to 1×1×1 mm voxels to avoid variations due to the use of scanning methods and equipment before extracting the radiomics features. The open-source Python package PyRadiomics (https://www.radiomics.io/py radiomics.html) was used for radiomics features extraction. The types of images included the original image and Wavelet and Laplacian of Gaussian (LogG, sigma 3.0) transform image based on the transformation of the original image. The radiomics features mainly included the following: 12 diagnostics, 19 first order, 17 shape, 24 gray level co-occurrence matrix (GLCM), 16 gray level size-zone matrix (GLSZM), 16 gray level run-length matrix (GLRLM), 5 neighborhood gray tone difference matrix (NGTDM), 14 neighboring gray level dependence matrix (GLDM), and Wavelet features and Laplacian of Gaussian features. As a result, a total of 2871 radiomics features were extracted from each ROI. A detailed description of the above radiomics features can be found at https://pyradiomics.readthedocs.io/en /latest/features.html. After feature extraction, all radiomics features were standardized using the z-score for further analysis.

### 2.3 Radiomics Features Selection and Radiomics Signature Construction

Radiomics features selection was performed on the training set. In order to select robust radiomics features for constructing radiomics signature, two feature selection procedures were performed for dimensionality reduction. First, we performed a two-sample t-test to select features that were significantly different between the MIBC and NMIBC groups. The features with significant differences between different groups (MIBC *vs*. NMIBC) were included in further analyses. Second, the least absolute shrinkage and selection operator (LASSO) regression was performed to select the radiomics features highly correlated with MIBC.

### 2.4 Establishment of Machine Learning Models

In this study, two commonly used machine learning methods for classification (logistic regression and SVC) were used to establish the models. Among them, the SVC used three different kernels to construct three models separately. The logistic regression solver was chosen as lbfgs, using L2 regularization and a C value of 1.0. The kernel function of SVC was linear, poly, and rbf, and the specific penalty coefficient C set for the tolerance of misclassified samples ranged from 0.0001 to 1000, num = 200. All models were trained using a five-fold cross-validation method to prevent overfitting. The complete construction process of the model is shown in Fig. (**2**).

### 2.5 Assessment of Models

The receiver operating characteristic curve (ROC) and area under the curve (AUC) value were used to evaluate the performance of the models. The accuracy, sensitivity, specificity, and F1-score were also calculated. To assess the calibration and clinical utility of the models, calibration curves and decision curve analysis were developed.

### 2.6 Statistical analysis

Continuous variables were presented as mean ± standard deviation (SD) and analyzed using either the two-sample t-test or the Mann-Whitney U test. Categorical variables were reported as frequencies and percentages, with comparisons made using the χ^2^ test or Fisher's exact test. Statistical analyses were conducted using IBM SPSS Statistics (version 25). A p value < 0.05 was considered statistically different.

## 3 RESULTS

### 3.1 Patient Characteristics

In this study, 148 patients were included and randomly assigned to the training set (n=118) and the testing set (n=30). The external testing set included 63 patients. All patients underwent transurethral resection. Table **2** summarizes the baseline characteristics of patients in the training and testing groups. There was no significant difference in age, gender, tumor size, and the number of lesions per patient between the two datasets.

### 3.2 Radiomics Features Selection

**Fig. (3):**
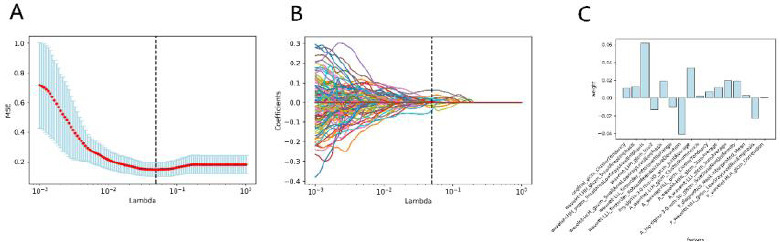
Texture feature selection using the least absolute shrinkage and selection operator (LASSO) binary logistic regression model. **A)** Tuning parameter (λ) selection in the LASSO model used 10-fold cross-validation according to the minimum criteria. The area under the receiver operating characteristic (AUC) curve was plotted *versus* log(λ). Dotted vertical lines were drawn at the optimal values by using the minimum criteria and 1 standard error of the minimum criteria (the 1-SE criteria). A log (λ) value of 0.053 was chosen according to 10-fold cross-validation. **B)** LASSO coefficient profiles of the texture features. A coefficient profile plot was produced against the log (λ) sequence. A vertical line was drawn at the value selected using 10-fold cross-validation, where optimal λ resulted in 15 non-zero coefficients. **C)** The feature importance based on the absolute value of coefficients at the current value of the log (λ).

First, 414 radiomics features were selected by employing a two-sample t-test. Second, the optimum parameter lambda (λ) was selected from the LASSO model using 10-fold cross-validation with the minimum mean square error (MSE) (Fig. **3A**). In order to filter the features, the coefficients of some features were downregulated to 0 by adjusting the weight parameters λ (Fig. **3B**). Consequently, the LASSO regression identified 15 features. Finally, 15 features were selected for radiomics signature construction. The features selected included 8 non-enhanced features, 5 arterial features, and 2 venous features. The weights of selected features are shown in Fig. (**3C**). We also calculated the Pearson correlation coefficient of the selected features and removed features with a correlation greater than 0.9. No strong correlation was found between the 15 features. The heatmap of the correlation values is shown in Fig. (**4**).

### 3.3 Model Building and Testing

**Fig. (5):**
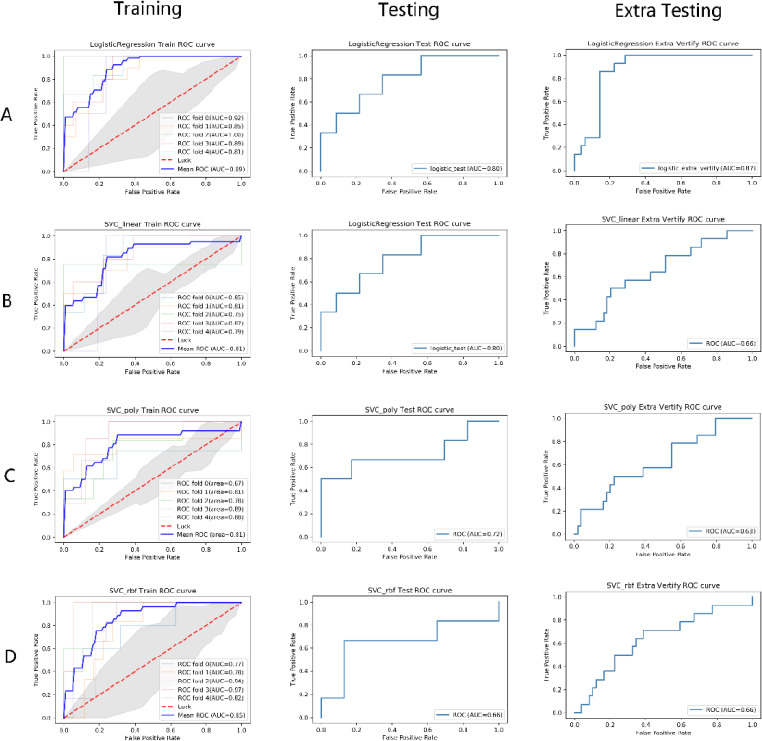
A comparison of the ROC curves of models built using four different machine learning methods on the training, testing, and external testing sets. In the training set, we used five-fold cross-validation and plotted ROC curves for each training session using colored thin lines, while the blue thick lines represent their average ROC curves. **A)** Logistic regression; **B)** support vector classification with linear kernel; **C)** support vector classification with poly kernel; **D)** support vector classification with rbf kernel.

The 15 radiomics features selected by LASSO regression were used for model construction. We built one logistic regression model and three SVC models with different kernel functions. The SVC three kernel functions included linear, poly, and rbf. The specific performances of all models in the training set, testing set, and external testing set are displayed in Table, and all ROC curves are depicted in (Fig. **5**). The logistic regression model exhibited the best prediction performance of MIBC. The result of the five-fold cross-validation in the training set indicated that the logistic regression model performed well with a mean AUC value of 0.89, and performed better than other models in the testing set and external testing set with an AUC value of 0.80 and 0.87; the diagnostic accuracy, precision, sensitivity, specificity, and F1-score of the model in the testing set and external testing set were 0.759, 0.826, 0.863, 0.729, 0.785, and 0.794, 0.755, 0.953, 0.720, and 0.809, respectively. The detailed results are shown in Fig. (**5**, Tables **3** and **4**). The calibration curves and decision curve analysis of all models are illustrated in Fig. (**6**). In the training set, the four models achieved similar predictive performance. However, in the test and validation sets, the model based on logistic regression performed the best for MIBC, being consistent with the ROC and AUC of models.

## 4 DISCUSSION

In this study, we preliminarily constructed machine learning models based on the radiomics features of CT images to predict the muscle-invasive status of bladder cancer. The best model showed good diagnostic performance for predicting preoperatively and non-invasively. In the training, testing, and external testing groups, the AUC values of this model were 0.89, 0.80, and 0.87, respectively.

The treatment and prognosis of bladder cancer largely depend on accurate staging and judgment of muscle invasion. The clinical staging represents non-muscle-invasive bladder cancer (NMIBC) as ≤ T1 stage, while muscle-invasive bladder cancer (MIBC) as ≥ T2 stage. Not only are the stages different, but the probability of NMIBC with a 5-year probability of progression is 45%, while the prognosis of MIBC is poor, with about 50% of patients experiencing metastasis within 2 years after radical cystectomy [16-18]. It has been observed that the definite clinical staging of muscle-invasive bladder cancer can determine the qualification of systemic treatment and/or radical cystectomy [19]. At present, clinical doctors determine the clinical staging based on the histopathological diagnosis of biopsy samples obtained through cystoscopy. Firstly, invasive surgery reduces patient acceptance of biopsy [20]. Secondly, relying on the experience of surgeons for cystoscopy tissue sampling can lower the quality of biopsy, and clinical staging may be underestimated [21, 22]. Clinical studies have shown that approximately 40% of MIBC patients are misdiagnosed as NMIBC after repeated sampling [23]. This study used four machine learning methods to establish the model, and the evaluation results of the model have been found to have high reliability and robustness. The proposed machine learning model may become an important supplement to pathological diagnosis and help clinical doctors to be more cautious in evaluating preoperative pathological biopsy results.

According to some previous large sample clinical studies, there is a certain gender difference between NMIBC and MIBC. Although the incidence rate of bladder cancer in men is higher than that in women, women are more likely to be diagnosed with MIBC than men at the time of onset, and the results after treatment are not good [1, 24]. Other studies have also shown women to have a higher proportion of MIBC patients, which may be attributed to their susceptibility to urinary tract infections and delayed early pathological examination and diagnosis [24]. However, there was no significant gender difference between NMIBC and MIBC in our sample, which could be attributed to the small sample size we studied. In addition, other baseline features in this study, such as age, tumor size, and number of lesions, were not statistically significant, which to some extent indicates the influence of baseline factors to be relatively small.

MRI is recognized as an important non-invasive tool to evaluate the muscle invasion status of bladder cancer. Previous studies have shown that the MRI-based Vesical Imaging Reporting and Data System (VI-RADS) can more accurately distinguish MIBC and NMIBC, and has good consistency among readers [25]. A large number of studies have also demonstrated the effectiveness of the VI-RADS system in the preoperative evaluation of the muscle invasion status of bladder cancer [26-28]. Therefore, the VI-RADS grading system is the most widely used method to determine the degree of muscle invasion of bladder cancer. In multi-parameter MRI images, DWI sequences have been repeatedly proven to be superior to T2WI sequences in reflecting heterogeneous differences between NMIBC and MIBC [2, 18], and DWI sequences play a significant role in the VI-RADS grading system. With the development of omics, radiation omics has been applied to MRI data to predict the muscle invasion status of bladder cancer. In 2017, Xu *et al*. [29] first predicted the muscle invasion status of bladder cancer through MRI using radiomics, and then carried out extensive research on the accurate differentiation between NMIBC and MIBC using the radiomics method involving multi-parameter MRI images, with the accuracy rate rising. Among them, the average accuracy of T2WI, DWI and two sequence combinations obtained by Xu *et al*. [30] was 79.63%, 81.37%, and 91.22%, respectively, and AUC was 88.28%, 88.84%, and 97.56%, respectively. The results were very satisfactory. Although many achievements have been made in MRI research, the population suitable for magnetic resonance imaging is still limited due to the many contraindications of MRI, such as long scanning time, metal implants, and claustrophobia. Although CT has limited image tissue resolution, it has fast scanning time and almost no contraindications. It can use isotropic images for multi-plane and three-dimensional reconstruction, which is beneficial for doctors to conduct preoperative evaluation and formulate surgical strategies. CT is an important supplement to MRI and an indispensable part of preoperative evaluation. Previously, all studies were based on one-phase CECT images (mostly venous phase), and the main purpose of the study was to perform pathological staging [11, 31, 32]. In this study, we innovatively combined the extraction of radiomics features from three-phase CECT images, which could obtain dynamic features while expanding the extractable radiomics feature values, providing richer basic data for feature selection. In order to ensure reliable and robust results, multiple machine learning methods were used to construct models, and the optimal AUC values under the ROC curve were obtained in the training, testing, and external testing groups, with values of 0.89,0.80 and 0.87, respectively, serving as an important supplement to clinical evaluation. This study demonstrated the ability of the CT radiomics model to predict the muscle invasion status of bladder cancer, being consistent with the study performed by Cui *et al*. [13]. This may provide useful computer-aided diagnostic tools for clinical decision-making.

In this work, we employed a range of machine learning approaches that are currently prevalent and effective in the field of bladder cancer. We compared four approaches, including logic regression and three different kernels of SVC; due to the small amount of data we used, in order to ensure that the training results did not overfit, we employed a five-fold cross-validation approach to train the models for all four methods and the model based on logic regression attained the best prediction performance. This result has been found to be consistent with the extensive validation in the computer vision field.

In this study, we also found that among the selected radiomics labels, 7 were in the non-enhancement scan, 5 in the arterial phase, and only 2 in the venous phase. In terms of radiomics, non-enhancement scan images still have very important application value, and their potential image features can provide important classification information. Arterial and venous phase images contribute more to classification than plain scan images, and may be more of a supplement. In this study, we also extracted the radiomics features based on wavelet transform and Laplace transform, which provided more abundant data for feature screening. In this study, 11 radiomics tags based on wavelet transform were reserved, which indicated that texture features after wavelet transform play an important role in classification. Further analysis showed the low-frequency component (L) in wavelet transform to account for a large proportion (Fig. **3C**). Therefore, the tissue resolution and structural information of the image may have contributed more to judging muscle infiltration of bladder cancer than image edge and contrast.

There were some limitations and shortcomings in this study. Firstly, this study was based on a limited sample size and lacked large external validation sets, which may pose issues in terms of generalizability. Further evaluation of the predictive model's practical clinical significance in predicting bladder cancer muscle invasion status requires a large sample size from multiple centers. Secondly, this study was retrospective and it may lead to certain biases. Prospective studies are needed to ensure minimal bias and provide more information on the progression of bladder cancer. Finally, in terms of diagnostic performance evaluation, the specificity of several machine learning models for diagnosis was not very high, which may be related to the small amount of data and may be improved by adding more clinically relevant information.

## CONCLUSION

In conclusion, this study has constructed an MCECT-based machine learning model to predict the muscle-invasive status of bladder cancer. This model exhibited fairly good diagnostic efficiency to differentiate MIBC and NMIBC of bladder cancer, which may provide a more powerful supplement for the clinical decision and management of bladder cancer.

## AUTHORS’ CONTRIBUTION

It is hereby acknowledged that all authors have accepted responsibility for the manuscript's content and consented to its submission. They have meticulously reviewed all results and unanimously approved the final version of the manuscript.

## Figures and Tables

**Fig. (1):**
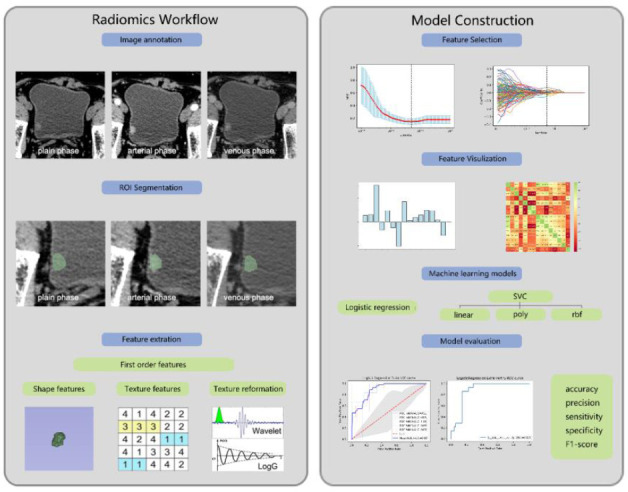
Radiomics and machine learning model construction workflow for predicting bladder cancer’s muscle-invasive status. The radiomics workflow includes image annotation, ROI segmentation, and feature extraction. The model construction workflow includes radiomics features selection, machine learning model construction, and model evaluation.

**Fig. (2):**
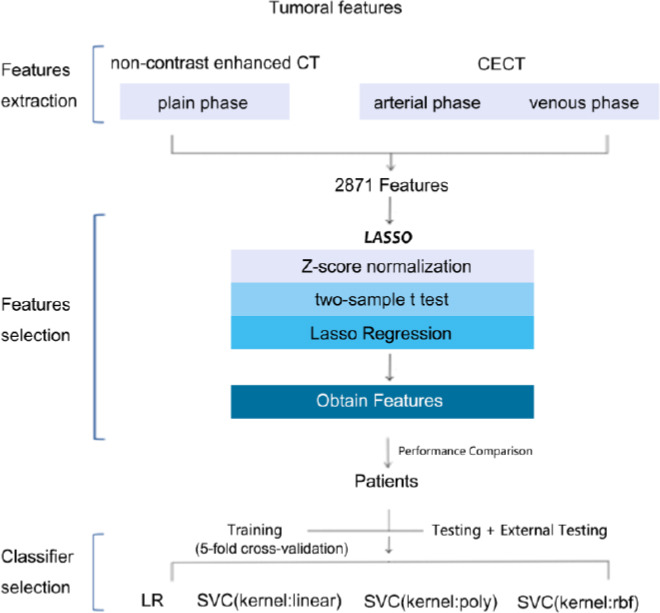
The detailed construction process of prediction models.

**Fig. (4):**
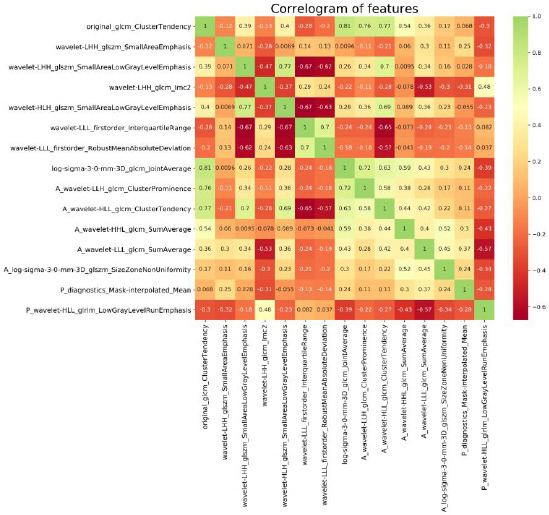
A heatmap of the correlation between the Pearson coefficients of the selected radiomics features. From the graph, we can see that the positive and negative correlations between the selected feature values did not exceed 90%, and all feature values were retained.

**Fig. (6):**
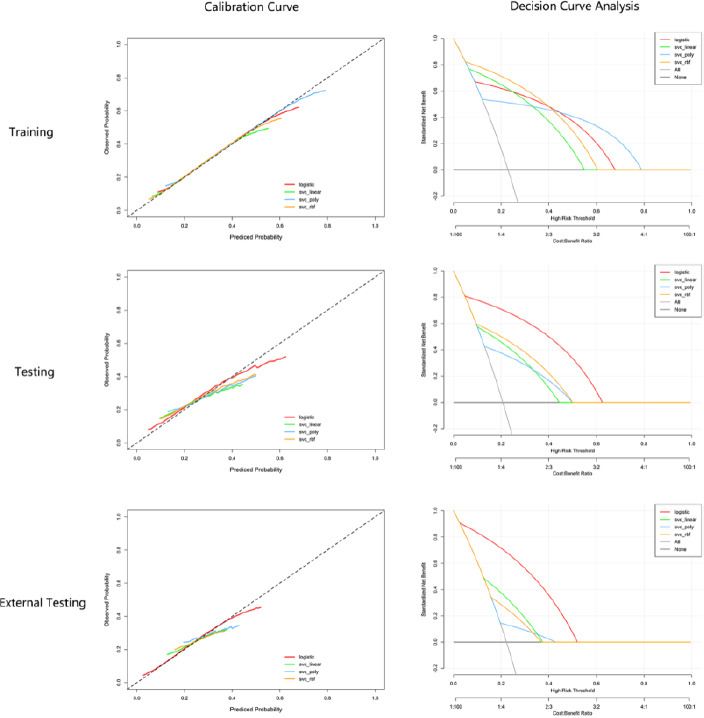
The calibration curves and decision curves for different models in the training, testing, and external testing sets.

**Table 1:** The detailed image acquisition scanning parameters.

**Acquired Parameters**	**Value**	
Equipment type	Discovery CT750HD	Somatom Definition Flash
Manufacturer	GE Healthcare	Siemens Healthineers
Tube voltage	120KV	120KV
Tube current	Smart mA 360 - 600	210
Scan type	Helical	Helical
FOV	40	50
Section thickness	1.0	1.0
Section intervals	1.0	1.0
Acquisition	64*0.6mm	128*0.6mm
Window width	200-250	200-250
Window level	30-50	30-50
Injection flow rate	3.5-4.5ml/s	3.5-4.5ml/s
Contrast agent dosage	80-100ml	80-100ml
Arterial phase delayed time	30-35s	30-35s
Venous phase delayed time	60-70s	60-70s

**Table 2:** Evaluation of the general information between patients in the training dataset and the test dataset.

	**Training group** **(n = 118)**	**Testing group** **(n = 30)**	**External** **testing group** **(n = 63)**	***X* ^2^/*t* value**	***P*-value**
Gender				0.069	0.793
Male	89	22	42		
Female	29	8	21		
Age (year)				0.061	0.805
≤60	48	11	24		
>60	70	19	39		
Tumor size (mm)	40.2 ± 19.0	35.6 ± 17.4	39 ± 16.6	1.201	0.232
The number of lesions				0.046	0.831
1	109	28	58		
2	9	2	5		
3	0	0	0		
Grade (pathology)				0.066	0.797
Low	68	18	35		
High	50	12	28		
Muscle invasion				0.002	0.963
Yes	28	9	14		
No	90	21	49		
Connective tissue invasion				3.081	0.079
Yes	56	10	25		
No	62	20	38		

**Table 3:** The AUC values of four different machine learning models on the training and test sets.

	AUC (%)		
	Training Group	Testing Group	External Testing Group
LR	89	80	87
SVC (linear)	81	75	66
SVC (poly)	81	72	63
SVC (rbf)	85	66	66

**Table 4:** Detailed metrics for evaluating the classification performance of four machine learning classifiers on the test set and the external test set.

Model	Dataset	Accuracy	Precision	Sensitivity	Specificity	F1-score
LR	T	0.759	0.826	0.863	0.729	0.785
	E	0.794	0.755	0.953	0.720	0.809
SVC (linear)	T	0.759	0.783	0.801	0.545	0.774
	E	0.683	0.694	0.872	0.475	0.706
SVC (poly)	T	0.793	0.870	0.869	0.501	0.763
	E	0.762	0.818	0.803	0.429	0.730
SVC (rbf)	T	0.793	0.826	0.804	0.501	0.703
	E	0.698	0.755	0.841	0.369	0.713

T: test set, E: external test set.

## Data Availability

The data and supportive information are available within the article.

## LIST OF ABBREVIATIONS

CECT: Contrast-enhanced computer tomography
MCECT: Multi-phase contrast-enhanced computer tomography
LASSO: Least absolute shrinkage and selection operator
ROC: Receiver operating characteristic
AUC: Area under the curve
MIBC: Muscle-invasive bladder cancer
NMIBC: Non-muscle-invasive bladder cancer
SVC: Support vector classification
ROI: Region of interest
VI-RADS: Vesical Imaging-Reporting and Data System
ML: Machine learning

## STANDARDS OF REPORTING

STROBE guidelines were followed.

## References

[1] Antoni S., Ferlay J., Soerjomataram I., Znaor A., Jemal A., Bray F. (2017). Bladder cancer incidence and mortality: A global overview and recent trends.. Eur. Urol..

[2] Wang H., Xu X., Zhang X., Liu Y., Ouyang L., Du P., Li S., Tian Q., Ling J., Guo Y., Lu H. (2020). Elaboration of a multisequence MRI-based radiomics signature for the preoperative prediction of the muscle-invasive status of bladder cancer: A double-center study.. Eur. Radiol..

[3] Dobruch J., Oszczudłowski M. (2021). Bladder cancer: Current challenges and future directions.. Medicina (Kaunas).

[4] Babjuk M., Burger M., Capoun O., Cohen D., Compérat E.M., Dominguez Escrig J.L., Gontero P., Liedberg F., Masson-Lecomte A., Mostafid A.H., Palou J., van Rhijn B.W.G., Rouprêt M., Shariat S.F., Seisen T., Soukup V., Sylvester R.J. (2022). European association of urology guidelines on non–muscle-invasive bladder cancer (TA, T1, and carcinoma *in situ*).. Eur. Urol..

[5] Brocklehurst A., Varughese M., Birtle A. (2023). Bladder preservation for muscle-invasive bladder cancer with variant histology.. Semin. Radiat. Oncol..

[6] Ucpinar B., Erbin A., Ayranci A., Caglar U., Alis D., Basal S., Sarilar O., Akbulut M.F. (2019). Prediction of recurrence in non-muscle invasive bladder cancer patients. Do patient characteristics matter?. J. Buon..

[7] Wong V.K., Ganeshan D., Jensen C.T., Devine C.E. (2021). Imaging and management of bladder cancer.. Cancers (Basel).

[8] Alfred Witjes J., Max Bruins H., Carrión A., Cathomas R., Compérat E., Efstathiou J.A., Fietkau R., Gakis G., Lorch A., Martini A., Mertens L.S., Meijer R.P., Milowsky M.I., Neuzillet Y., Panebianco V., Redlef J., Rink M., Rouanne M., Thalmann G.N., Sæbjørnsen S., Veskimäe E., van der Heijden A.G. (2024). European association of urology guidelines on muscle-invasive and metastatic bladder cancer: Summary of the 2023 guidelines.. Eur. Urol..

[9] Li J., Ma S., Wu D., Zhang Z., Chen Y., Liu B., Li C., Jia H. (2025). CT-based radiomics and cluster analysis for the prediction of local progression in stage I NSCLC patients treated with microwave ablation.. iScience.

[10] Akcay A., Yagci A.B., Celen S., Ozlulerden Y., Turk N.S., Ufuk F. (2021). VI-RADS score and tumor contact length in MRI: A potential method for the detection of muscle invasion in bladder cancer.. Clin. Imaging.

[11] Zhang G., Xu L., Zhao L., Mao L., Li X., Jin Z., Sun H. (2020). CT-based radiomics to predict the pathological grade of bladder cancer.. Eur. Radiol..

[12] Wang H., Hu D., Yao H., Chen M., Li S., Chen H., Luo J., Feng Y., Guo Y. (2019). Radiomics analysis of multiparametric MRI for the preoperative evaluation of pathological grade in bladder cancer tumors.. Eur. Radiol..

[13] Cui Y., Sun Z., Liu X., Zhang X., Wang X. (2022). CT-based radiomics for the preoperative prediction of the muscle-invasive status of bladder cancer and comparison to radiologists’ assessment.. Clin. Radiol..

[14] Zheng Z., Xu F., Gu Z., Yan Y., Xu T., Liu S., Yao X. (2021). Combining multiparametric MRI radiomics signature with the vesical imaging-reporting and data system (VI-RADS) score to preoperatively differentiate muscle invasion of bladder cancer.. Front. Oncol..

[15] Chen G., Fan X., Wang T., Zhang E., Shao J., Chen S., Zhang D., Zhang J., Guo T., Yuan Z., Tang H., Yu Y., Chen J., Wang X. (2023). A machine learning model based on MRI for the preoperative prediction of bladder cancer invasion depth.. Eur. Radiol..

[16] Sylvester R.J., van der Meijden A.P.M., Oosterlinck W., Witjes J.A., Bouffioux C., Denis L., Newling D.W.W., Kurth K. (2006). Predicting recurrence and progression in individual patients with stage Ta T1 bladder cancer using EORTC risk tables: A combined analysis of 2596 patients from seven EORTC trials.. Eur. Urol..

[17] Sherif A., Jonsson M.N., Wiklund N.P. (2007). Treatment of muscle-invasive bladder cancer.. Expert Rev. Anticancer Ther..

[18] Huang X., Wang X., Lan X., Deng J., Lei Y., Lin F. (2022). The role of radiomics with machine learning in the prediction of muscle-invasive bladder cancer: A mini review.. Front. Oncol..

[19] Hensley P.J., Panebianco V., Pietzak E., Kutikov A., Vikram R., Galsky M.D., Shariat S.F., Roupret M., Kamat A.M. (2022). Contemporary staging for muscle-invasive bladder cancer: Accuracy and limitations.. Eur. Urol. Oncol..

[20] Mariappan P., Zachou A., Grigor K.M. (2010). Detrusor muscle in the first, apparently complete transurethral resection of bladder tumour specimen is a surrogate marker of resection quality, predicts risk of early recurrence, and is dependent on operator experience.. Eur. Urol..

[21] Svatek R.S., Shariat S.F., Novara G., Skinner E.C., Fradet Y., Bastian P.J., Kamat A.M., Kassouf W., Karakiewicz P.I., Fritsche H.M., Izawa J.I., Tilki D., Ficarra V., Volkmer B.G., Isbarn H., Dinney C.P. (2011). Discrepancy between clinical and pathological stage: External validation of the impact on prognosis in an international radical cystectomy cohort.. BJU Int..

[22] Turker P., Bostrom P.J., Wroclawski M.L., van Rhijn B., Kortekangas H., Kuk C., Mirtti T., Fleshner N.E., Jewett M.A., Finelli A., Kwast T.V., Evans A., Sweet J., Laato M., Zlotta A.R. (2012). Upstaging of urothelial cancer at the time of radical cystectomy: Factors associated with upstaging and its effect on outcome.. BJU Int..

[23] Yanagisawa T., Kawada T., von Deimling M., Bekku K., Laukhtina E., Rajwa P., Chlosta M., Pradere B., D’Andrea D., Moschini M., Karakiewicz P.I., Teoh J.Y.C., Miki J., Kimura T., Shariat S.F. (2024). Repeat transurethral resection for non–muscle-invasive bladder cancer: An updated systematic review and meta-analysis in the contemporary era.. Eur. Urol. Focus.

[24] Dobruch J., Daneshmand S., Fisch M., Lotan Y., Noon A.P., Resnick M.J., Shariat S.F., Zlotta A.R., Boorjian S.A. (2016). Gender and bladder cancer: A collaborative review of etiology, biology, and outcomes.. Eur. Urol..

[25] Barchetti G., Simone G., Ceravolo I., Salvo V., Campa R., Del Giudice F., De Berardinis E., Buccilli D., Catalano C., Gallucci M., Catto J.W.F., Panebianco V. (2019). Multiparametric MRI of the bladder: Inter-observer agreement and accuracy with the Vesical Imaging-Reporting and Data System (VI-RADS) at a single reference center.. Eur. Radiol..

[26] Wang H., Luo C., Zhang F., Guan J., Li S., Yao H., Chen J., Luo J., Chen L., Guo Y. (2019). Multiparametric MRI for bladder cancer: Validation of VI-RADS for the detection of detrusor muscle invasion.. Radiology.

[27] Metwally M.I., Zeed N.A., Hamed E.M., Elshetry A.S.F., Elfwakhry R.M., Alaa Eldin A.M., Sakr A., Aly S.A., Mosallam W., Ziada Y.M.A., Balata R., Harb O.A., Basha M.A.A. (2021). The validity, reliability, and reviewer acceptance of VI-RADS in assessing muscle invasion by bladder cancer: A multicenter prospective study.. Eur. Radiol..

[28] Ueno Y., Tamada T., Takeuchi M., Sofue K., Takahashi S., Kamishima Y., Urase Y., Kido A., Hinata N., Harada K., Fujisawa M., Miyaji Y., Murakami T. (2021). VI-RADS: Multiinstitutional multireader diagnostic accuracy and interobserver agreement study.. AJR Am. J. Roentgenol..

[29] Xu X., Liu Y., Zhang X., Tian Q., Wu Y., Zhang G., Meng J., Yang Z., Lu H. (2017). Preoperative prediction of muscular invasiveness of bladder cancer with radiomic features on conventional MRI and its high-order derivative maps.. Abdom. Radiol. (N.Y.).

[30] Xu X., Zhang X., Tian Q., Wang H., Cui L.B., Li S., Tang X., Li B., Dolz J., Ayed I., Liang Z., Yuan J., Du P., Lu H., Liu Y. (2019). Quantitative identification of nonmuscle-invasive and muscle-invasive bladder carcinomas: A multiparametric MRI radiomics analysis.. J. Magn. Reson. Imaging.

[31] Lin P., Wen D., Chen L., Li X., Li S., Yan H., He R., Chen G., He Y., Yang H. (2020). A radiogenomics signature for predicting the clinical outcome of bladder urothelial carcinoma.. Eur. Radiol..

[32] Cha K.H., Hadjiiski L.M., Cohan R.H., Chan H.P., Caoili E.M., Davenport M.S., Samala R.K., Weizer A.Z., Alva A., Kirova-Nedyalkova G., Shampain K., Meyer N., Barkmeier D., Woolen S., Shankar P.R., Francis I.R., Palmbos P. (2019). Diagnostic accuracy of CT for prediction of bladder cancer treatment response with and without computerized decision support.. Acad. Radiol..

